# Photosensitivity, corneal scarring and developmental delay: Xeroderma Pigmentosum in a tropical country

**DOI:** 10.1186/1757-1626-1-254

**Published:** 2008-10-20

**Authors:** James Halpern, Bryan Hopping, Joshua M Brostoff

**Affiliations:** 1 Specialty Registrar in Dermatology, University Hospital of North Staffordshire, UK; 2 Medical Student, Touro University College of Osteopathic Medicine, CA, USA; 3 Supervising Physician, Hospitalito Atitlàn, Santiago Atitlàn, Guatemala

## Abstract

We present the case of an 8 year-old girl in a developing country with significant corneal scarring and multiple cutaneous skin lesions in sun-exposed areas. Neuro-developmental delay had been present since 3 months of age, and taken as a whole the consensus was that this clinical picture was consistent with Xeroderma Pigmentosum (XP). We highlight the difficulties encountered due to the lack of diagnostic and treatment modalities for this child, and offer a brief review of XP, including emerging treatments that show potential.

## Case report

We present the case of an 8 year-old girl who presented to the out-patient department of a small hospital in Santiago Atitlàn, Guatemala, after maternal concern regarding progressive ocular lesions. The family was of Mayan heritage and spoke Tz'utujil and a small amount of Spanish. The history was unusual: since the age of 3 months the patient had suffered with persistent developmental delay as well as the appearance of multiple pigmented papular lesions on her face, neck, and forearms. Over time these lesions had enlarged and become progressively more numerous and raised, although were confined to sun-exposed areas. In the last year her mother had noted gradually enlarging corneal lesions bilaterally.

Following normal vaginal delivery at home with no pre-natal care, development had been limited to social smiling, sufficient fine motor skills to feed herself, infrequent speech of only single words, and an unsteady gait. Although a generally happy and smiling child, the patient had never been self-caring with regard to dressing and toileting, and was still wearing nappies.

She had 2 normal siblings, and there was no family history or other past medical history of note. The mother denied any consanguinity in the patient's recent lineage.

**Figure 1:**
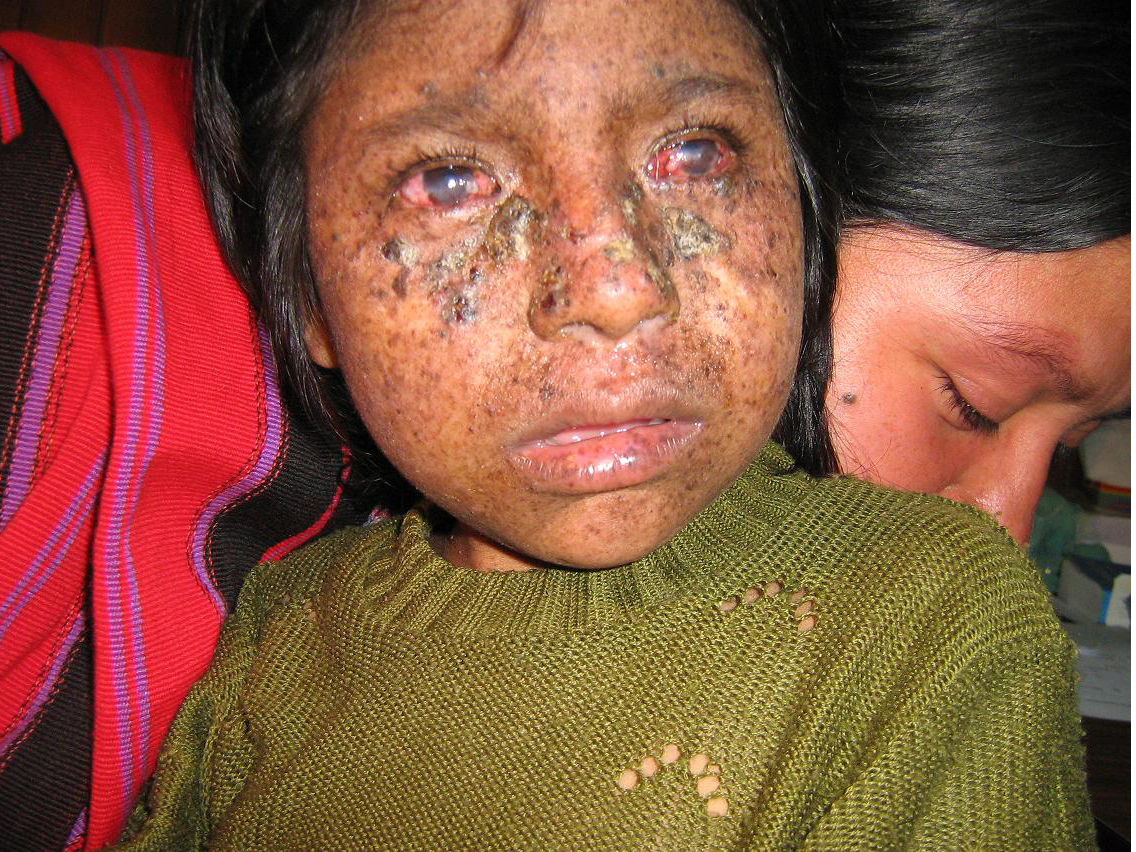
**Frontal image of face, showing large hyperkeratotic lesions with some induration suspicious of actinic keratosis and early squamous cell carcinoma.** Also numerous hyper-pigmented lentigos and xerosis on the entire front of the face. Marked corneal scarring and injection is evident.

**Figure 2:**
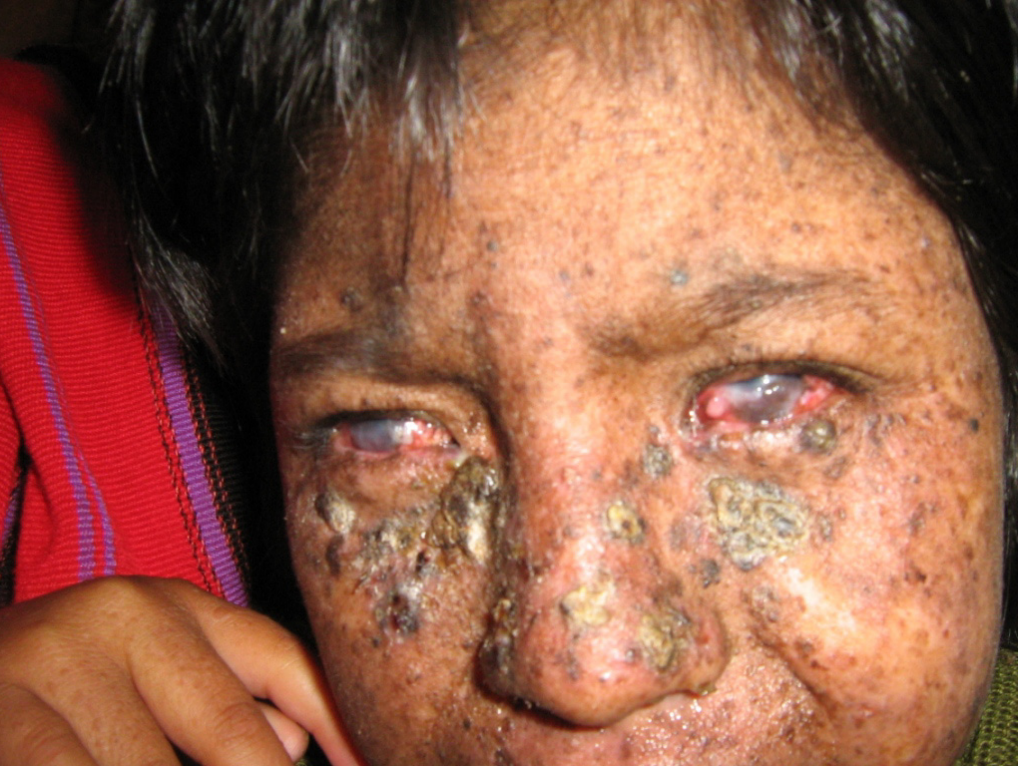
Close-up of the face shows the corneal scarring and ulceration, as well as the diffuse hyperkeratotic lesions.

**Figure 3:**
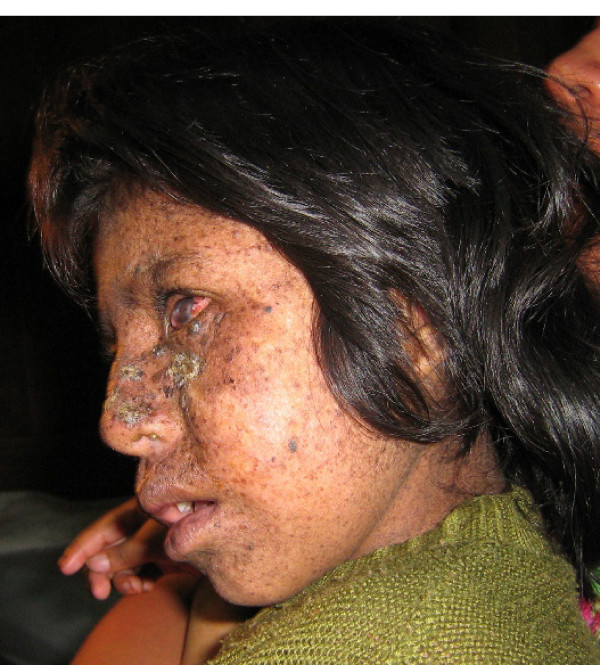
This left lateral view of the face shows the diffuse nature of the hyperpigmented lentigos on the face and neck, as well as demonstrating again the corneal damage and para-nasal hyperkeratosis.

On examination the patient was smiling and playful, and appeared well-nourished. Large hyperkeratotic lesions were present on the cheeks and nose with some induration suspicious of actinic keratosis and early squamous cell carcinoma. There were also numerous hyper-pigmented lentigos and xerosis limited to sun-exposed sites. Marked corneal scarring was evident bilaterally (Figures 1, 2, 3). There was no evidence of anaemia or overt signs of vitamin deficiencies such as rickets, and the mother and siblings appeared well nourished.

Physical examination of the heart, lungs and abdomen was unremarkable. There was mild choreoathetosis of the right arm, and gait was broad-based and grossly ataxic. Eye movements exhibited rolling nystagmus, but were able to track large objects. The patient was constantly reaching to the ground, trying to pick up things and put them in her mouth – either a neuro-developmental problem, or possibly indicative of pica. Unfortunately no laboratory testing was available for further investigation.

Given the cutaneous, neurological and ocular features a presumptive diagnosis of Xeroderma Pigmentosum was made. As facilities for pathological diagnosis were not available an opinion was sought from international colleagues. There was consensus that the clinical features were those of a photosensitive dermatoses with Xeroderma Pigmentosum being the most likely given the striking cutaneous features.

The patient was offered referral to ophthalmology and paediatric specialists in Guatemala City, which was declined. The patient has subsequently been lost to follow up.

## Discussion

Xeroderma Pigmentosum (XP) is a rare genetic disorder that occurs worldwide in all races and ethnic groups. First described by Hebra and Kaposi in 1874 the disorder is characterised by marked photosensitivity and premature onset of all major types of skin cancer [1].

From an early age patients are sensitive to even minimal sun exposure developing erythema, vesicles and oedema. By the age of two years solar lentigos, xerosis and pigmentation occur. Later in childhood dysplastic and neoplastic lesions occur with the development of actinic keratosis, keratocanthoma, basal cell carcinoma, squamous cell carcinoma and malignant melanoma [1]. In one study the median age for development of malignant melanoma was 8 years of age [2]. Ocular complications are nearly as common as skin lesions with keratitis progressing to corneal opacification, loss of eyelashes, ectropion, entropion and benign and malignant lesions of the cornea and eyelids. Neurological complications occur in approximately 30% of cases and can be severe [1,3].

XP is usually inherited in an autosomal recessive manner with phenotypically normal heterozygotes. There are at least seven different subtypes (complementation groups A-G) as well as XP variants. Various rare forms occur in combination with other disorders such as Cockayne's Syndrome [1]. 80% of patients have classical XP where there is a defect in the initiation of DNA nucleotide excision repair after UV induced damage. In XP variant the defect is found in post-replication or daughter-strand repair.

The diagnosis of XP is considered when a young patient presents with marked photosensitivity, xerosis and multiple pigmented lesions. Phototesting may be performed showing reduced minimal erythema dose in the 290 to 340 nm range [4]. Although phototesting is widely available in the developed world it is neither sensitive or specific for XP. The diagnostic test of choice is time consuming, highly specialised and expensive: cultured fibroblasts are extracted from a skin biopsy, fused with fibroblasts from known XP lines and exposed to UV irradiation. If the subsequent DNA repair is defective, the XP complementation group may be identified from the fused XP line used. Recent developments include the cloning of all XP genes, so the complementation group can also be determined by using recombinant retroviral vectors.

Guatemala has no centralized health records for prevalence of XP or subtype analysis, and to date there has been only one published report of a cluster of cases of the group C complementation form in a single rural Guatemalan community [5]. Of the sub-types, XP-V occurs at a later age and is less debilitating, while XP-C and XP-E sufferers do not usually have neurological complications. XP-A or XP-D are the most likely genotypes to have caused the phenotypic presentation of this patients.

Management of XP consists of early and rigorous photoprotection with sun avoidance, sunscreens and appropriate clothing. Experimental treatments with topical DNA repair enzymes and oral retinoids are showing promise for the future [6,7].

There are particular challenges when a child with XP grows up in a tropical environment as illustrated by this case. The geographical and cultural bars to medical facilities led to a significant delay in diagnosis for this patient. This has resulted in years of photodamage resulting in the patients striking appearance and undoubted limited lifespan. Culturally the patient would have spent most of the day outdoors in the equatorial high UV exposure environment. Even if a medical opinion was sought earlier in life we must consider the lack of local specialists and the high cost of the specialised diagnostic tests needed. Perhaps most importantly the language barrier limited the medical team's ability to perform genetic counselling vital to the parents and local tribes understanding of the condition.

## Abbreviations

DNA: Deoxyribonucleic Acid; UV: ultra-violet; XP: Xeroderma Pigmentosum.

## Competing interests

The authors declare that they have no competing interests.

## Authors' contributions

JMB and BH wrote the case report. Discussion written by JH and JMB.

## Consent

Written informed consent was obtained from the patient's mother for publication of this case report and accompanying images. A copy of the written consent is available for review by the Editor-in-Chief of this journal.
